# Draft Genome Sequences of *Streptococcus bovis* Strains ATCC 33317 and JB1

**DOI:** 10.1128/genomeA.01012-14

**Published:** 2014-10-09

**Authors:** Faiza H. Benahmed, Gopal R. Gopinath, Heather Harbottle, Michael A. Cotta, Yan Luo, Carol Henderson, Plona Teri, Daniel Soppet, Mark Rasmussen, Terence R. Whitehead, Maureen Davidson

**Affiliations:** a U.S. Food and Drug Administration, Center for Veterinary Medicine, Division of Animal and Food Microbiology, Laurel, Maryland, USA; b SAIC-Frederick National Laboratory for Cancer Research, Frederick, Maryland, USA; c U.S. Food and Drug Administration, Center for Food Safety and Applied Nutrition, Office of Applied Research and Safety Assessment, Division of Virulence Assessment, Laurel, Maryland, USA; d U.S. Department of Agriculture, ARS, National Center for Agricultural Utilization Research, Bioenergy Research Unit, Peoria, Illinois, USA; e U.S. Food and Drug Administration, Center for Food Safety and Applied Nutrition, Office of Analytics and Outreach, Division of Public Health Informatics and Analytics, College Park, Maryland, USA

## Abstract

We report the draft genome sequences of *Streptococcus bovis* strain ATCC 33317 (CVM42251) isolated from cow dung and strain JB1 (CVM42252) isolated from a cow rumen in 1977. The strains were sequenced using the Genome Sequencer FLX 454 system. The genome sizes are approximately 2 Mb and 2.2 Mb, respectively.

## GENOME ANNOUNCEMENT

*Streptococcus bovis* is a common inhabitant of the rumen of domestic livestock; it has been implicated as a causative agent of lactic acidosis in the rumen (1, 2) and as a cause of human disease (3, 4). Strains of *S. bovis* recently were reclassified into various new species and subtypes, primarily due to interest in the potential relationships of *S. bovis* to endocarditis and colon cancer in humans (5, 6). A previous work indicated that ruminal and human strains of *S. bovis* are different at the genetic level (7). We now report the draft genome sequence of the *S. bovis* strain ATCC 33317 and ruminal isolate JB1.

*In vitro* antimicrobial susceptibility testing was performed according to the standard National Antimicrobials Resistance Monitoring System (NARMS) protocol (8) using the CMV3AGPF Gram-positive (Trek/Thermo Diagnostics Systems; Cleveland, OH) panel of antimicrobials and the veterinary pathogen panel BOPO6F (Trek). Both strains ATCC 33317 and JB1 are resistant to enrofloxacin (MIC, 2 µg/ml) and sulfadimethoxine (MIC, >256 µg/m) antimicrobials.

Genomic DNA from each strain was extracted from overnight cultures, as previously described (9, 10). DNA libraries were constructed according to the Roche protocol with the GS Rapid library preparation kit. The libraries were then sequenced using the Genome Sequencer FLX 454 system (Roche, Branford, CT) and the GS FLX Titanium sequencing kit XLR70, according to the manufacturer’s recommended protocol, to obtain a coverage of >10-fold. The Roche Newbler software version 2.3 (Roche, Branford, CT) was used for the *de novo* assembly of the paired-end reads, resulting in 17 contigs (strain ATCC 33317) and 45 contigs (strain JB1). The draft genome sequences of *S. bovis* strains ATCC 33317 (CVM42251) and JB1 (CVM42252) consist of 1,842,663 bp and 1,955,516 bp, respectively, with *N*_50_ contig sizes of 463,296 bp and 150,226 bp, respectively. The draft genomes have an overall G+C content of 37.2% for strain ATCC 33317 and 37.6% for strain JB1. Contigs of >200 bp were annotated using the NCBI Prokaryotic Genomes Annotation Pipeline (PGAP) version 2.1 (http://www.ncbi.nlm.nih.gov/genomes/static/Pipeline.html) and were deposited at DDBJ/EMBL/GenBank. A total of 1,748 (strain ATC33317) and 1,909 (strain JB1) predicted coding sequences (CDSs) were detected. In addition to the CDSs, the annotation revealed 46 RNA genes, including 44 tRNA and 2 rRNA genes for strain ATCC 33317, and 37 RNA genes, including 34 tRNA and 3 rRNA genes for strain JB1.

### Nucleotide sequence accession numbers.

The draft genome sequences for these two *S. bovis* strains have been deposited at DDBJ/EMBL/GenBank under accession numbers AUZG00000000 and AUZH00000000.
